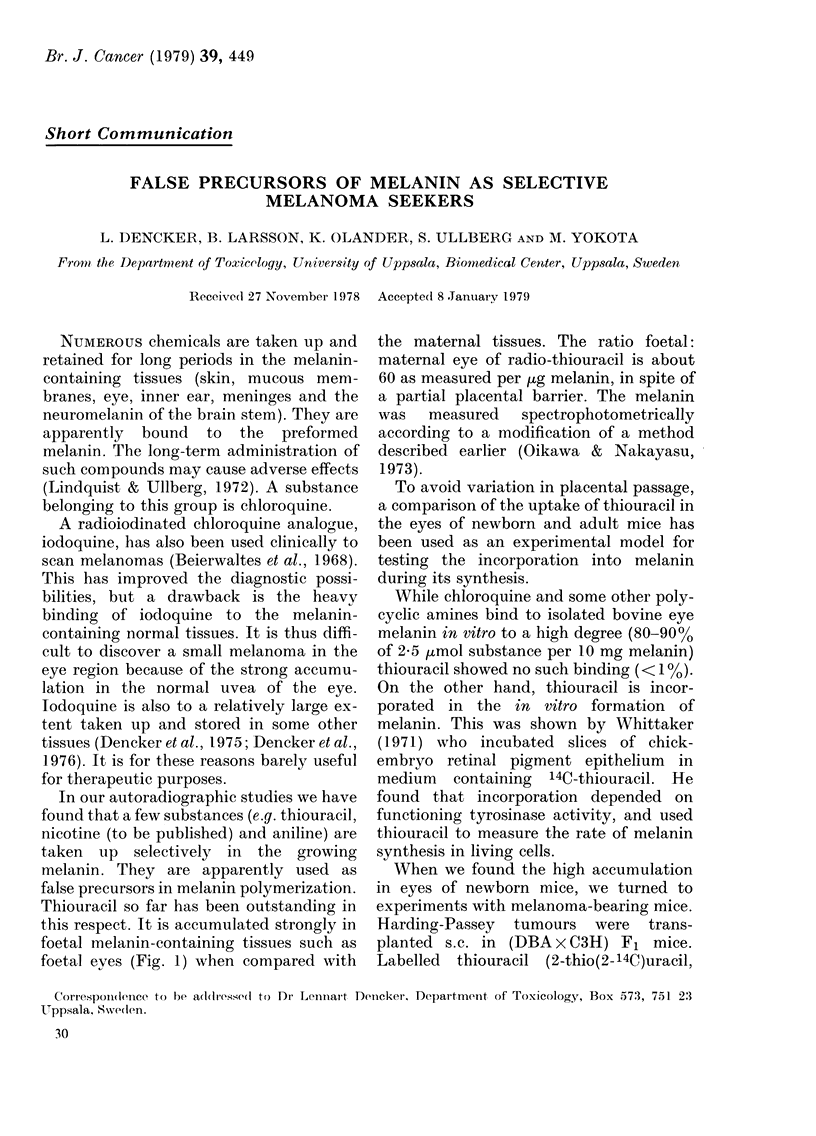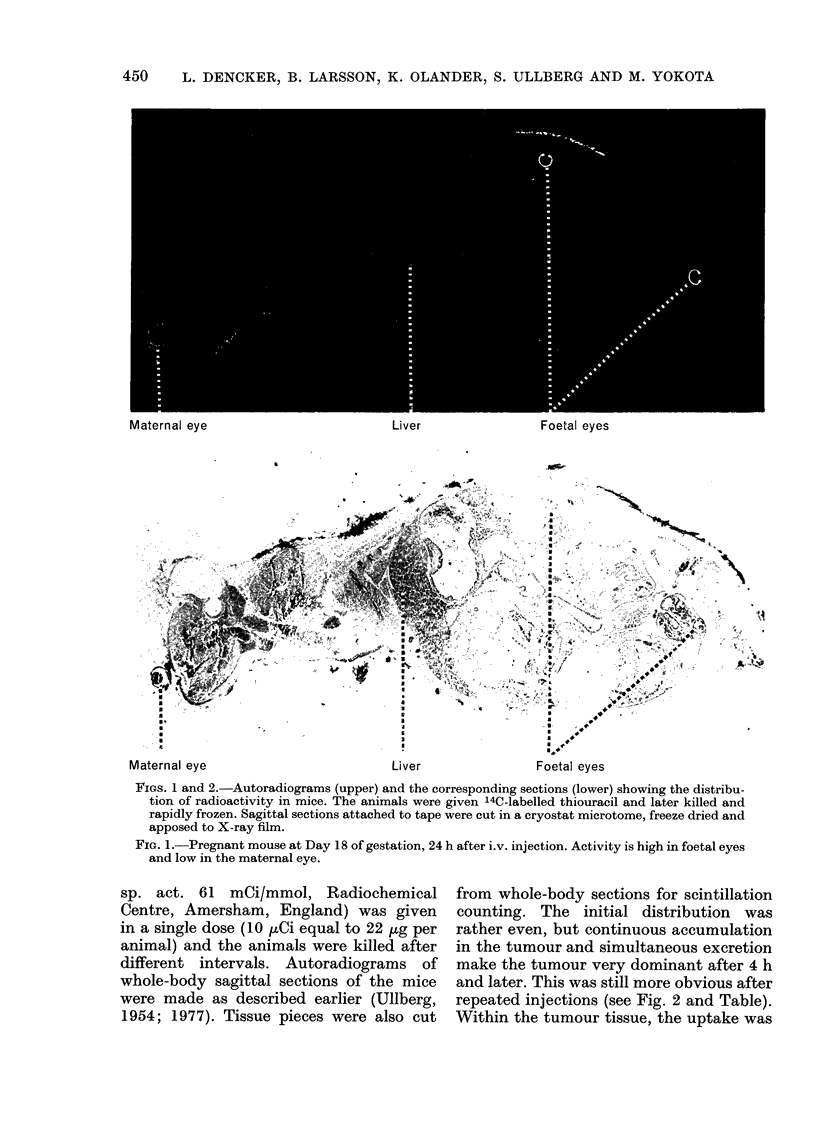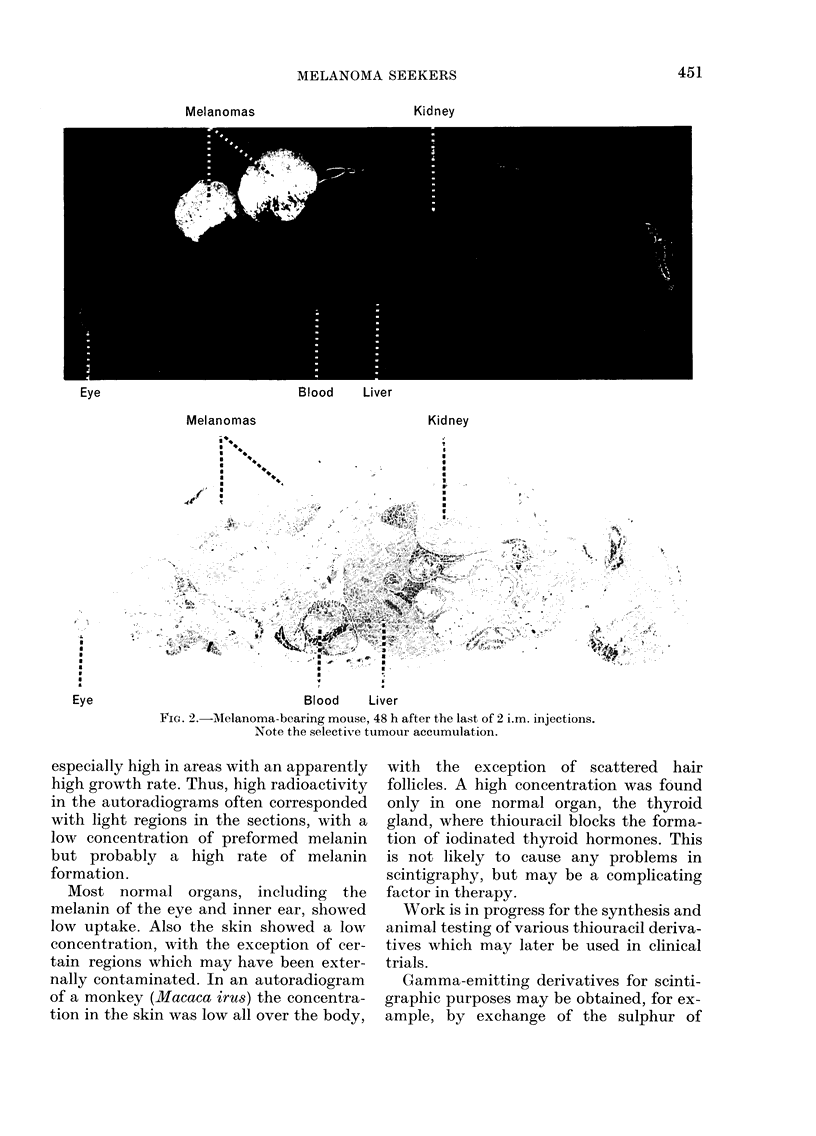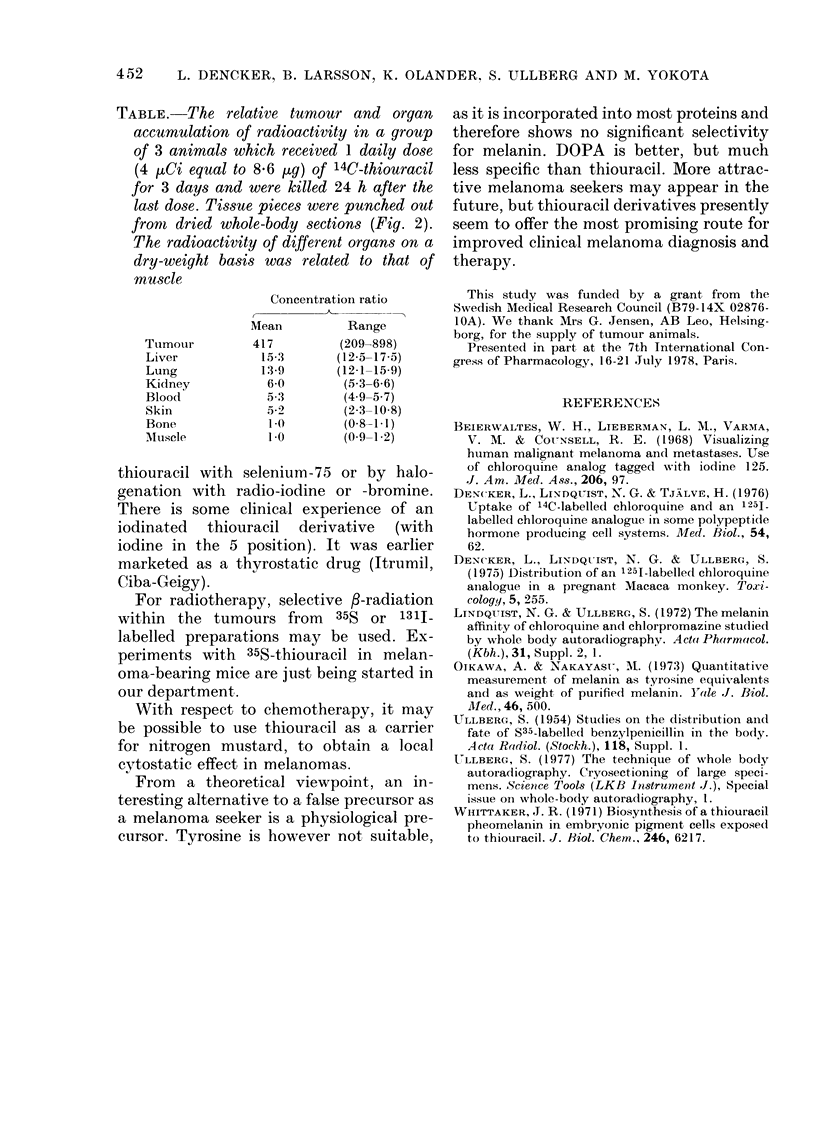# False precursors of melanin as selective melanoma seekers.

**DOI:** 10.1038/bjc.1979.80

**Published:** 1979-04

**Authors:** L. Dencker, B. Larsson, K. Olander, S. Ullberg, M. Yokota

## Abstract

**Images:**


*Br. J. Cancer* (1979) **39**, 449

***Short Communication***

### FALSE PRECURSORS OF MELANIN AS SELECTIVE MELANOMA SEEKERS

L. DENCKER, B. LARSSON, K. OLANDER, S. ULLBERG AND M. YOKOTA

*From the Department of Toxicology, University of Uppsala, Biomedical Center, Uppsala, Sweden*

Received 27 November 1978 Accepted 8 January 1979

NUMEROUS chemicals are taken up and retained for long periods in the melanin-containing tissues (skin, mucous membranes, eye, inner ear, meninges and the neuromelanin of the brain stem). They are apparently bound to the preformed melanin. The long-term administration of such compounds may cause adverse effects (Lindquist & Ullberg, 1972). A substance belonging to this group is chloroquine.

A radioiodinated chloroquine analogue, iodoquine, has also been used clinically to scan melanomas (Beierwaltes *et al.*, 1968). This has improved the diagnostic possibilities, but a drawback is the heavy binding of iodoquine to the melanin-containing normal tissues. It is thus difficult to discover a small melanoma in the eye region because of the strong accumulation in the normal uvea of the eye. Iodoquine is also to a relatively large extent taken up and stored in some other tissues (Dencker *et al.*, 1975; Dencker *et al.*, 1976). It is for these reasons barely useful for therapeutic purposes.

In our autoradiographic studies we have found that a few substances (*e.g.* thiouracil, nicotine (to be published) and aniline) are taken up selectively in the growing melanin. They are apparently used as false precursors in melanin polymerization. Thiouracil so far has been outstanding in this respect. It is accumulated strongly in foetal melanin-containing tissues such as foetal eyes (Fig. 1) when compared with the maternal tissues. The ratio foetal: maternal eye of radio-thiouracil is about 60 as measured per $\mu$g melanin, in spite of a partial placental barrier. The melanin was measured spectrophotometrically according to a modification of a method described earlier (Oikawa & Nakayasu, 1973).

To avoid variation in placental passage, a comparison of the uptake of thiouracil in the eyes of newborn and adult mice has been used as an experimental model for testing the incorporation into melanin during its synthesis.

While chloroquine and some other polycyclic amines bind to isolated bovine eye melanin *in vitro* to a high degree (80–90% of 2·5 $\mu$mol substance per 10 mg melanin) thiouracil showed no such binding (<1%). On the other hand, thiouracil is incorporated in the *in vitro* formation of melanin. This was shown by Whittaker (1971) who incubated slices of chick-embryo retinal pigment epithelium in medium containing $^{14}$C-thiouracil. He found that incorporation depended on functioning tyrosinase activity, and used thiouracil to measure the rate of melanin synthesis in living cells.

When we found the high accumulation in eyes of newborn mice, we turned to experiments with melanoma-bearing mice. Harding-Passey tumours were transplanted s.c. in (DBA$\times$C3H) $F_1$ mice. Labelled thiouracil (2-thio(2-$^{14}$C)uracil,

Correspondence to be addressed to Dr Lennart Dencker, Department of Toxicology, Box 573, 751 23 Uppsala, Sweden.

FIGS. 1 and 2.—Autoradiograms (upper) and the corresponding sections (lower) showing the distribution of radioactivity in mice. The animals were given $^{14}C$-labelled thiouracil and later killed and rapidly frozen. Sagittal sections attached to tape were cut in a cryostat microtome, freeze dried and apposed to X-ray film.

FIG. 1.—Pregnant mouse at Day 18 of gestation, 24 h after i.v. injection. Activity is high in foetal eyes and low in the maternal eye.

sp. act. 61 mCi/mmol, Radiochemical Centre, Amersham, England) was given in a single dose (10 $\mu$Ci equal to 22 $\mu$g per animal) and the animals were killed after different intervals. Autoradiograms of whole-body sagittal sections of the mice were made as described earlier (Ullberg, 1954; 1977). Tissue pieces were also cut from whole-body sections for scintillation counting. The initial distribution was rather even, but continuous accumulation in the tumour and simultaneous excretion make the tumour very dominant after 4 h and later. This was still more obvious after repeated injections (see Fig. 2 and Table). Within the tumour tissue, the uptake was

FIG. 2.—Melanoma-bearing mouse, 48 h after the last of 2 i.m. injections. Note the selective tumour accumulation.

especially high in areas with an apparently high growth rate. Thus, high radioactivity in the autoradiograms often corresponded with light regions in the sections, with a low concentration of preformed melanin but probably a high rate of melanin formation.

Most normal organs, including the melanin of the eye and inner ear, showed low uptake. Also the skin showed a low concentration, with the exception of certain regions which may have been externally contaminated. In an autoradiogram of a monkey (*Macaca irus*) the concentration in the skin was low all over the body, with the exception of scattered hair follicles. A high concentration was found only in one normal organ, the thyroid gland, where thiouracil blocks the formation of iodinated thyroid hormones. This is not likely to cause any problems in scintigraphy, but may be a complicating factor in therapy.

Work is in progress for the synthesis and animal testing of various thiouracil derivatives which may later be used in clinical trials.

Gamma-emitting derivatives for scintigraphic purposes may be obtained, for example, by exchange of the sulphur of

TABLE.—*The relative tumour and organ accumulation of radioactivity in a group of 3 animals which received 1 daily dose (4 μCi equal to 8·6 μg) of $^{14}$C-thiouracil for 3 days and were killed 24 h after the last dose. Tissue pieces were punched out from dried whole-body sections (Fig. 2). The radioactivity of different organs on a dry-weight basis was related to that of muscle*

| | Concentration ratio | |
|---|---|---|
| | Mean | Range |
| Tumour | 417 | (209–898) |
| Liver | 15·3 | (12·5–17·5) |
| Lung | 13·9 | (12·1–15·9) |
| Kidney | 6·0 | (5·3–6·6) |
| Blood | 5·3 | (4·9–5·7) |
| Skin | 5·2 | (2·3–10·8) |
| Bone | 1·0 | (0·8–1·1) |
| Muscle | 1·0 | (0·9–1·2) |

thiouracil with selenium-75 or by halogenation with radio-iodine or -bromine. There is some clinical experience of an iodinated thiouracil derivative (with iodine in the 5 position). It was earlier marketed as a thyrostatic drug (Itrumil, Ciba-Geigy).

For radiotherapy, selective β-radiation within the tumours from $^{35}$S or $^{131}$I-labelled preparations may be used. Experiments with $^{35}$S-thiouracil in melanoma-bearing mice are just being started in our department.

With respect to chemotherapy, it may be possible to use thiouracil as a carrier for nitrogen mustard, to obtain a local cytostatic effect in melanomas.

From a theoretical viewpoint, an interesting alternative to a false precursor as a melanoma seeker is a physiological precursor. Tyrosine is however not suitable, as it is incorporated into most proteins and therefore shows no significant selectivity for melanin. DOPA is better, but much less specific than thiouracil. More attractive melanoma seekers may appear in the future, but thiouracil derivatives presently seem to offer the most promising route for improved clinical melanoma diagnosis and therapy.

This study was funded by a grant from the Swedish Medical Research Council (B79-14X 02876-10A). We thank Mrs G. Jensen, AB Leo, Helsingborg, for the supply of tumour animals.

Presented in part at the 7th International Congress of Pharmacology, 16-21 July 1978, Paris.

### REFERENCES

BEIERWALTES, W. H., LIEBERMAN, L. M., VARMA, V. M. & COUNSELL, R. E. (1968) Visualizing human malignant melanoma and metastases. Use of chloroquine analog tagged with iodine 125. *J. Am. Med. Ass.*, **206**, 97.

DENCKER, L., LINDQUIST, N. G. & TJÄLVE, H. (1976) Uptake of $^{14}$C-labelled chloroquine and an $^{125}$I-labelled chloroquine analogue in some polypeptide hormone producing cell systems. *Med. Biol.*, **54**, 62.

DENCKER, L., LINDQUIST, N. G. & ULLBERG, S. (1975) Distribution of an $^{125}$I-labelled chloroquine analogue in a pregnant Macaca monkey. *Toxicology*, **5**, 255.

LINDQUIST, N. G. & ULLBERG, S. (1972) The melanin affinity of chloroquine and chlorpromazine studied by whole body autoradiography. *Acta Pharmacol.* (*Kbh.*), **31**, Suppl. 2, 1.

OIKAWA, A. & NAKAYASU, M. (1973) Quantitative measurement of melanin as tyrosine equivalents and as weight of purified melanin. *Yale J. Biol. Med.*, **46**, 500.

ULLBERG, S. (1954) Studies on the distribution and fate of $S^{35}$-labelled benzylpenicillin in the body. *Acta Radiol.* (*Stockh.*), **118**, Suppl. 1.

ULLBERG, S. (1977) The technique of whole body autoradiography. Cryosectioning of large specimens. *Science Tools* (*LKB Instrument J.*), Special issue on whole-body autoradiography, 1.

WHITTAKER, J. R. (1971) Biosynthesis of a thiouracil pheomelanin in embryonic pigment cells exposed to thiouracil. *J. Biol. Chem.*, **246**, 6217.